# An accurate, precise method for general labeling of extracellular vesicles

**DOI:** 10.1016/j.mex.2015.08.002

**Published:** 2015-09-10

**Authors:** Warren D. Gray, Adam J. Mitchell, Charles D. Searles

**Affiliations:** a Division of Cardiology, Emory University School of Medicine, Atlanta, GA, United States; b Atlanta Veterans Affairs Medical Center, Decatur, GA, United States

**Keywords:** Extracellular vesicles, Calcein AM, Flow cytometry

## Abstract

Extracellular, membrane vesicles (microvesicles, exosomes) are secreted by cells and may serve as mediators of intercellular communication. Methods for detecting them by flow cytometry have included the use of agents that fluorescently stain vesicle membrane, or fluorescent antibodies that target specific cell-of-origin antigens. However, these methods may falsely detect cell debris or require prior cell-of-origin knowledge. Here, we demonstrate the suitability of calcein AM for detection of intact extracellular vesicles (EVs) by flow cytometry.

• Calcein AM is non-fluorescent until it passively enters EVs, after which it is activated and becomes fluorescent and EV-impermeant.
• Permeabilized/lysed EVs label positive with antibodies and lipophilic membrane stain, whereas no labeling was observed with calcein. In contrast to methods that use antibodies or membrane stains, calcein AM allows for the differentiation between intact EVs and debris.
• Calcein AM can be used for detection of intact EVs from numerous cell types.

## Graphical abstract

**Figure:**
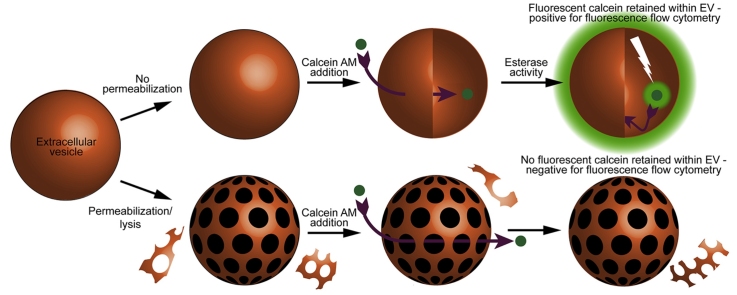

## Method details

### Rationale

Extracellular vesicles (EVs), including microvesicles/microparticles and exosomes, have been of interest to the scientific community for the roles they play in physiological and pathological processes, and their potential for therapeutic application [1], [2]. They range from 30 nm to 3 μm in diameter and typically consist of a phospholipid bilayer encompassing bioactive molecules, such as proteins and nucleic acids. The detection and analysis of extracellular vesicles by flow cytometry has been a challenge, as staining with non-specific membrane dyes or conjugated antibodies against specific cell-of-origin surface markers fail to discriminate between intact vesicles and debris. Furthermore, use of antibodies alone for the detection of EVs limits the study of EVs to required prior knowledge of the cells of origin.

An emerging strategy for flow cytometry-based analysis of extracellular vesicles is to label with a membrane-permeant molecule that is non-fluorescent until it is activated by intra-vesicular enzymes. Examples include 5-carboxyfluorescein diacetate-acetoxymethyl ester and calcein AM (an acetoxymethyl derivate of the fluorescent molecule calcein). Upon hydrolysis of the acetoxymethyl ester moieties by esterases, the fluorescent carboxyfluorescein and calcein are relatively membrane impermeant. These molecules have been used extensively for live cell detection [3], [4], [5] and tracking [6], as well as assessment of leakage from bioengineered liposomes [7]. A few studies have reported the use of calcein AM to label EVs, including exosomes [8], [9] and microvesicles [10], [11], [12], for detection in flow cytometry and microscopy. However, these studies did not present confirmatory evidence that fluorescence activation indicated only intact EVs, or that disrupted EV and EV debris were not labeled.

Here, we describe a simple and highly sensitive, accurate, and precise flow cytometry-based method that uses calcein AM to detect and quantify EVs from various sources. Furthermore, we provide evidence that this method can distinguish between intact vesicles and disrupted vesicles. Such a strategy for identifying intact EVs may prove valuable in evaluating the stability of vesicles, especially in reference to vesicular tolerance of temperature, freezing/thawing cycles, enzymatic activity, the extracellular environment, and changes in pH, osmolarity, and time. We have found the method to be widely applicable; we have observed that calcein AM allows for the detection of MVs from at least two species (human and mouse), multiple cell types (endothelial cells, platelets, red blood cells, and leukocytes), from cultured cells in various states (healthy, serum-starved, stimulated with TNF-α, or treated with caspase or rho-associated protein kinase inhibitors) and in the circulation of humans in various states of health (young, elderly, healthy, or those with cardiovascular disease, peripheral artery disease, or having experienced acute myocardial infarction).

Extracellular vesicles were isolated via serial centrifugation from three sources: stored red blood cells (RBCs); human plasma; and media of cultured human aortic endothelial cells (HAECs) (Lonza, cat. CC-2535). Packed RBCs (1 mL) were centrifuged at 1200 × *g* for 15 min, after which supernatant was removed (200 μL) and centrifuged at 800 × *g* for 10 min to remove residual RBCs. Red blood cell EVs were pelleted by centrifugation of the supernatant (180 μL) at 16,100 × *g* for 20 min. Platelet-depleted human plasma (50 μL) was diluted into 0.2 μm-filtered PBS (1.5 mL) and centrifuged at 16,100 × *g* for 20 min to pellet plasma EVs. HAECs were cultured for 24 h with complete culture media (Lonza, cat. CC-3162) that had been filtered (0.2 μm filter) to remove incidental EVs from supplemented bovine serum. The conditioned media (20 mL) was centrifuged at 800 × *g* for 10 min to remove cell debris, and the supernatant was subsequently centrifuged at 16,100 × *g* to pellet HAEC EVs.

**Fig. 1:**
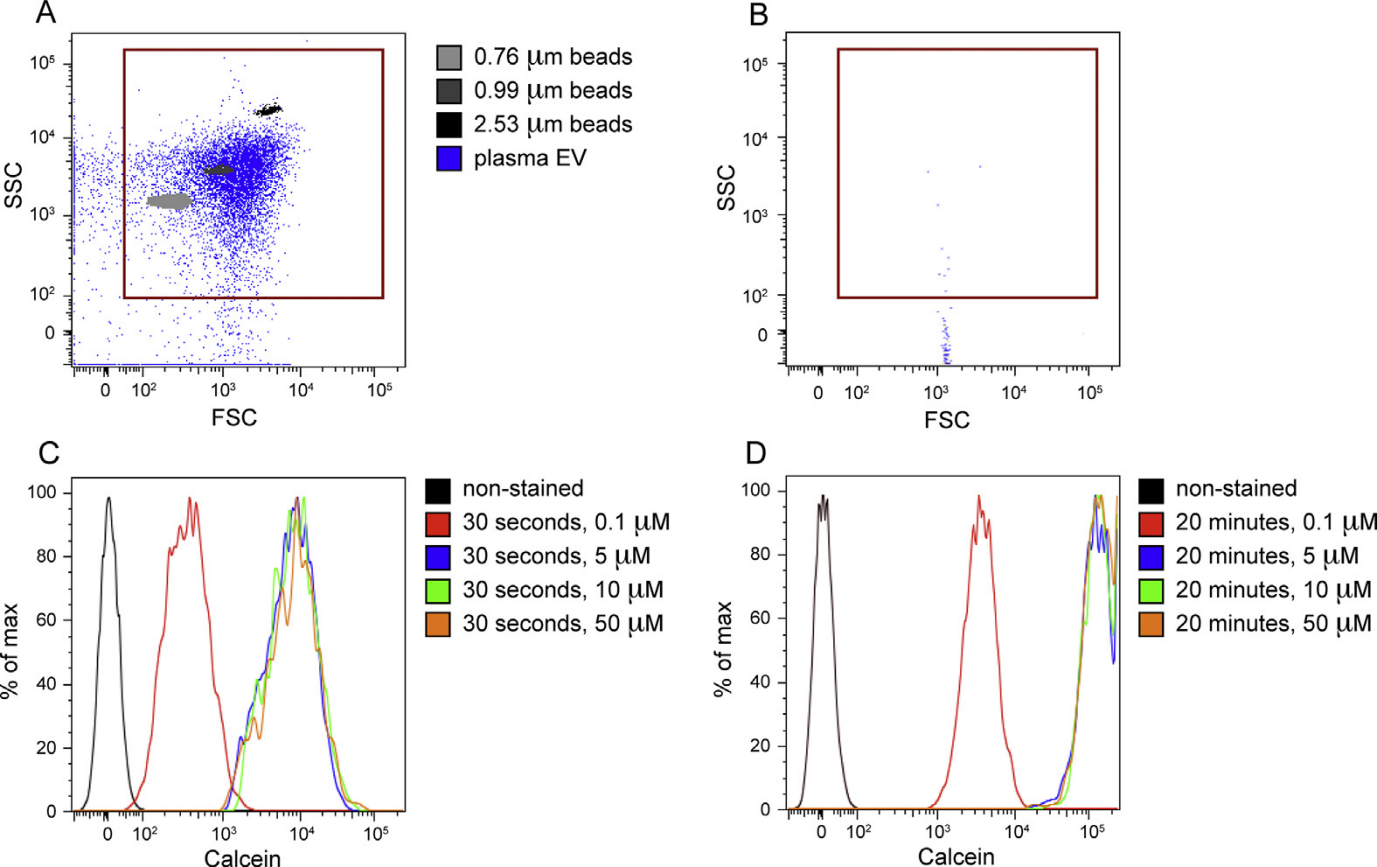
Forward and side scatter comparison of calibration beads with plasma EV gated events (A). Events in the red gate were considered EV candidates and subsequently gated for calcein fluorescence. Background noise in filtered PBS was minimal (B). Overlaid histograms of treatment groups, where the *x*-axis is calcein fluorescence intensity and the *y*-axis is the percentage of max; within each treatment group, the binned events are normalized to the maximum number of events. Fluorescently labeled plasma EVs were detected after 30 s of incubation with calcein AM (C); maximum fluorescence was observed after 20 min incubation with calcein AM (D).

Calcein AM (Life Technologies, cat. C3100MP) was solubilized in DMSO to produce a 100x solution (1 mM). This stock solution was diluted in filtered PBS to a final concentration of 10 μM. Extracellular vesicle pellets from the three sources were resuspended in 100 μL calcein AM working solution and incubated at 37 °C for 20 min to allow for intra-vesicular esterase activity to render the calcein AM fluorescent. The labeled EV suspensions were diluted with 200 μL PBS before data acquisition by flow cytometry (calcein excitation_max_495 nm/emission_max_516 nm, common fluorescein isothiocyanate channel) (Becton Dickinson, LSRII). A FSC/SSC dot plot compares gated events (EV candidates) with size calibration beads (Bangs Laboratories, Inc., cat. 832 and 833) (Fig. 1A). Gated events (Fig. 1A) were then selected for fluorescence above the background autofluorescence of non-stained EV candidates. The conversion of calcein AM in plasma MVs was apparent after 30 s of incubation (Fig. 1C). We used 20 min incubation with 10 μM calcein AM as standard conditions for reaching maximum fluorescence of different EVs (Fig. 1D).

**Fig. 2:**
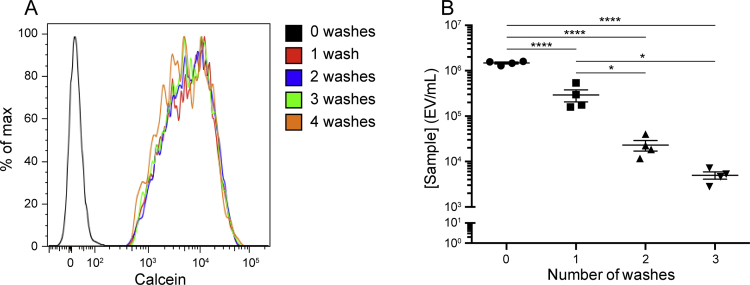
Overlaid fluorescence intensity histogram, normalized for each treatment group. Washing calcein AM-labeled plasma EVs did not appreciably change fluorescence intensity (A). Washing plasma EVs resulted in a significant loss of EVs with each wash (B). *p < 0.05, ****p < 0.0001, ANOVA followed by Tukey post-test. *n* = 4 for each treatment group.

Filtered PBS (200 μL) was then added to the suspended fluorescent EVs (300 μL final volume) before analysis by flow cytometry. To confirm that fluorescence was due to intra-vesicular calcein rather than free calcein, labeled EVs were subjected to multiple washes with PBS. The fluorescence spectrum did not shift appreciably after washing, indicating that fluorescence was not due to free activated calcein, which could lead to false detection of non-vesicular events (Fig. 2A). In fact, washing EVs caused significant loss of events (Fig. 2B) so the numbers of washing cycles were minimized, and EVs were labeled with calcein AM directly before flow cytometry processing. Concentrations of EVs were calculated by ratiometric comparison with Flow-Check Fluorospheres (Beckman Coulter, Inc., cat. 6605359).

### Validation Approach 1 – Sizing of isolated EVs

**Fig. 3:**
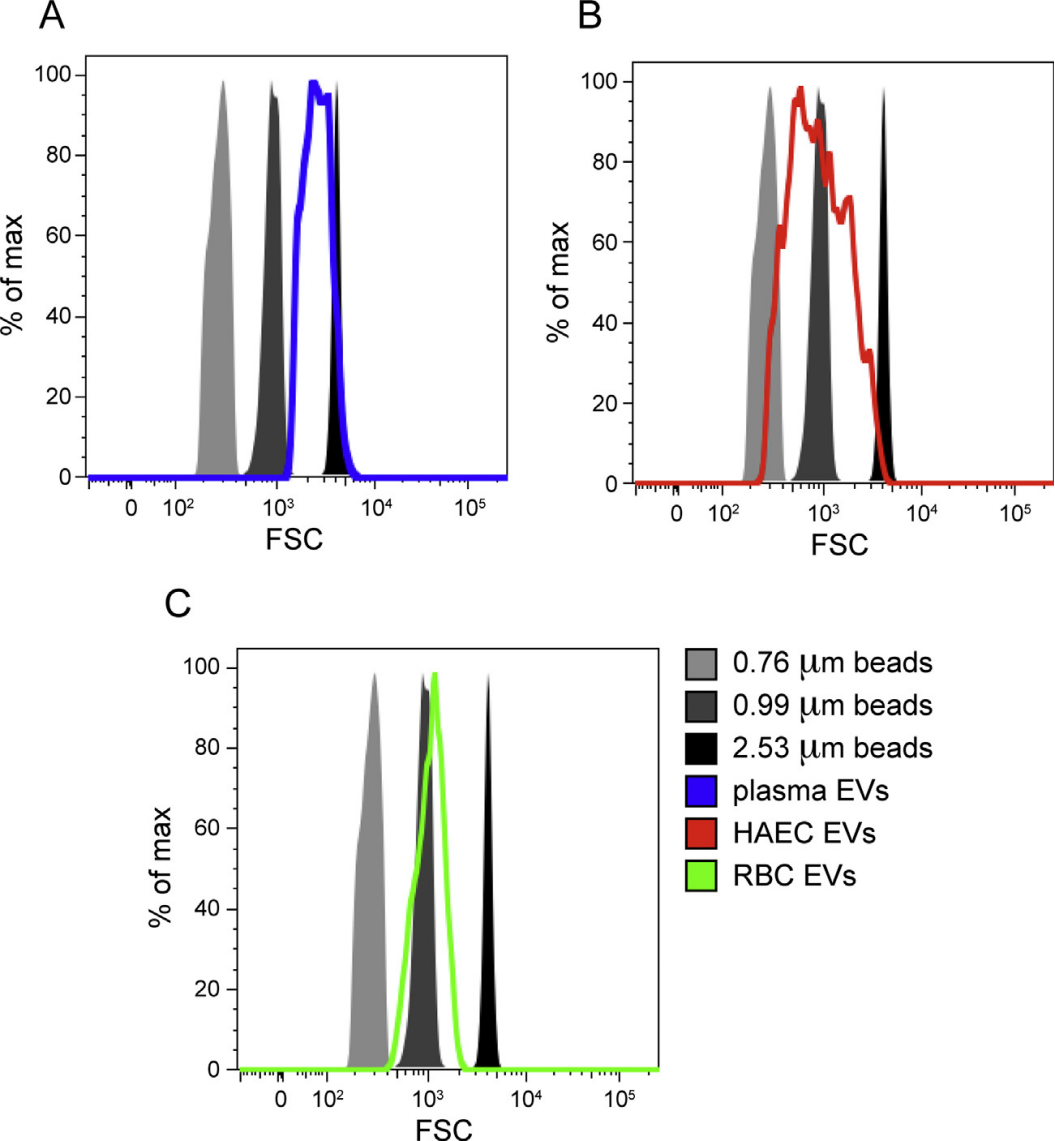
Sizing of plasma (A), HAEC (B), and RBC (C) EVs indicated a range from 0.76 μm to 2.53 μm.

To confirm that the isolated events were EV-sized, we acquired events in the flow cytometry forward scatter channel for the plasma, HAEC, and RBC EVs, as well as 0.76, 0.99, and 2.53 μm size calibration beads. Comparison to the size calibration beads indicated that the gated events were within the size range of EVs (Fig. 3).

### Validation Approach 2 – Permeabilization and general membrane staining

To demonstrate that calcein AM labels intact EVs-but not membrane fragments or other debris-we permeabilized the plasma, RBC, and HAEC EVs with two different agents: Triton X-100 (Sigma-Aldrich, cat. T-8787) and saponin (Sigma-Aldrich, cat. 47036-50G-F). Triton X-100 is a nonionic surfactant that non-specifically permeabilizes lipid membrane bilayers and lyses cells. Saponin is a plant-derived amphipathic glycoside that complexes with cholesterol to permeabilize membrane bilayers. Permeabilization would allow for esterase and calcein AM leakage from the EVs, and we hypothesized that we would see an inverse relationship between the concentration of the permeabilizing agent and calcein fluorescence intensity. To track the permeabilization and lysis of the EVs, we co-stained with the non-specific membrane dye, PKH26 (excitation_max_551 nm/emission_max_567 nm, common phycoerythrin (PE) channel) (Sigma-Aldrich, cat. MINI26-1KT).

After isolation, EVs were resuspended in varying concentrations of 0.2 μm-filtered Triton-X 100 (0%, 0.001%, 0.01%, 0.1%, and 1%) or saponin (0 mg/mL, 1 μg/mL, 10 μg/mL, 100 μg/mL, or 1 mg/mL) for 10 min or 20 min, respectively. The permeabilized EV suspensions were then 2× diluted in filtered PBS and centrifuged at 16,100 × *g* for 20 min to remove residual agents. The pellets were then resuspended in 100 μL of 10 μM calcein AM as before for 20 min at 37 °C. To prevent PKH26 micelle formation, physiologic salts in the PBS were removed by pelleting the calcein AM-labeled EVs (16,100 × *g*, 20 min) and resuspending the pellet in Diluent C (Sigma-Aldrich, provided with PKH26 dye) before addition of the PKH26 dye (2 μM final concentration). After 5 min incubation at room temperature, filtered PBS was added to the EV suspensions (300 μL final volume) before flow cytometry processing. Single stains of calcein AM and PKH26 on non-permeabilized EVs, along with an non-stained control, were prepared for compensation controls.

**Fig. 4:**
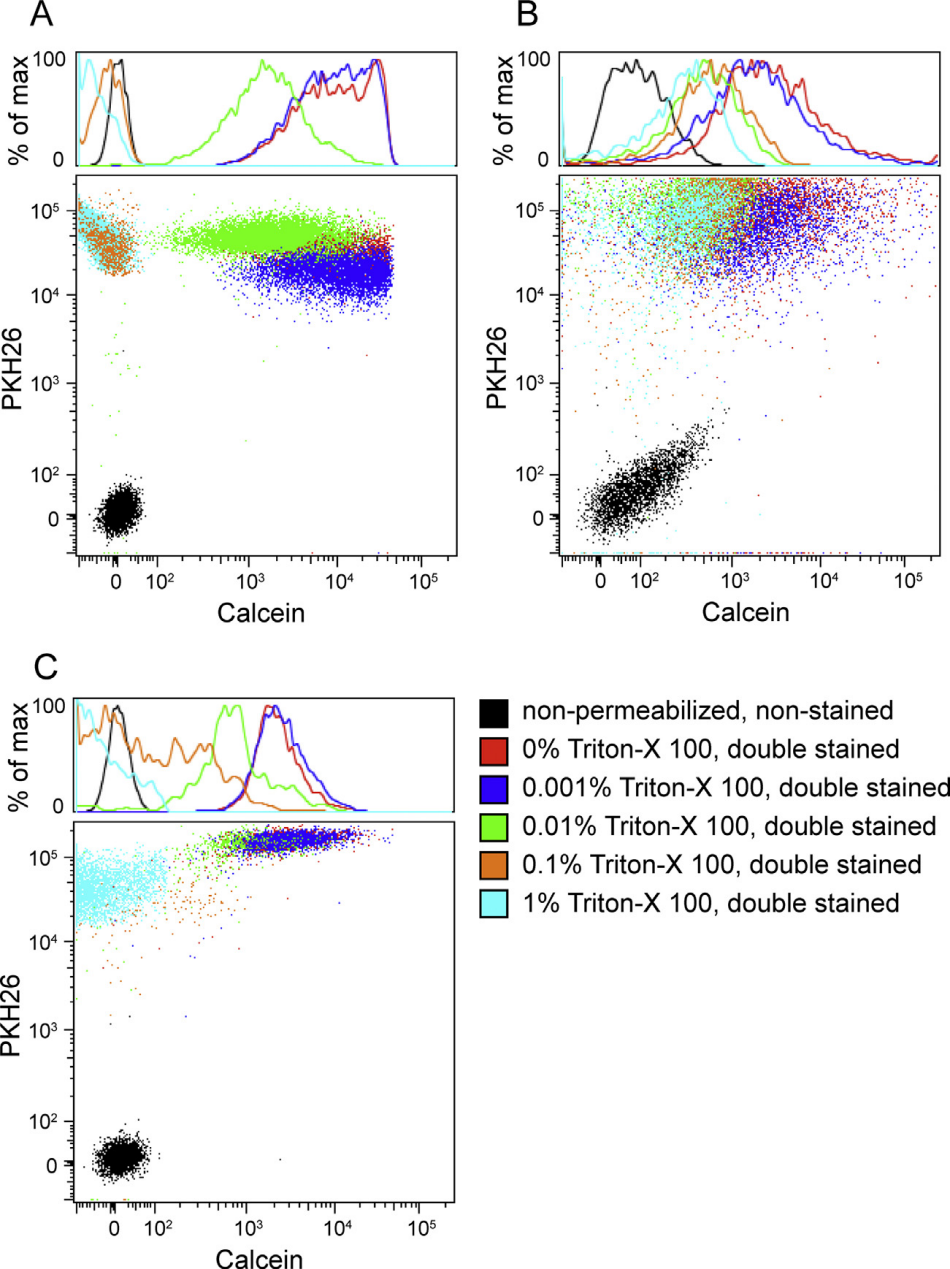
Pre-permeabilization with Triton X-100 rendered the permeabilized plasma (A), HAEC (B), and RBC (C) EVs unable to activate or retain calcein AM to the same degree as intact EVs.

**Fig. 5:**
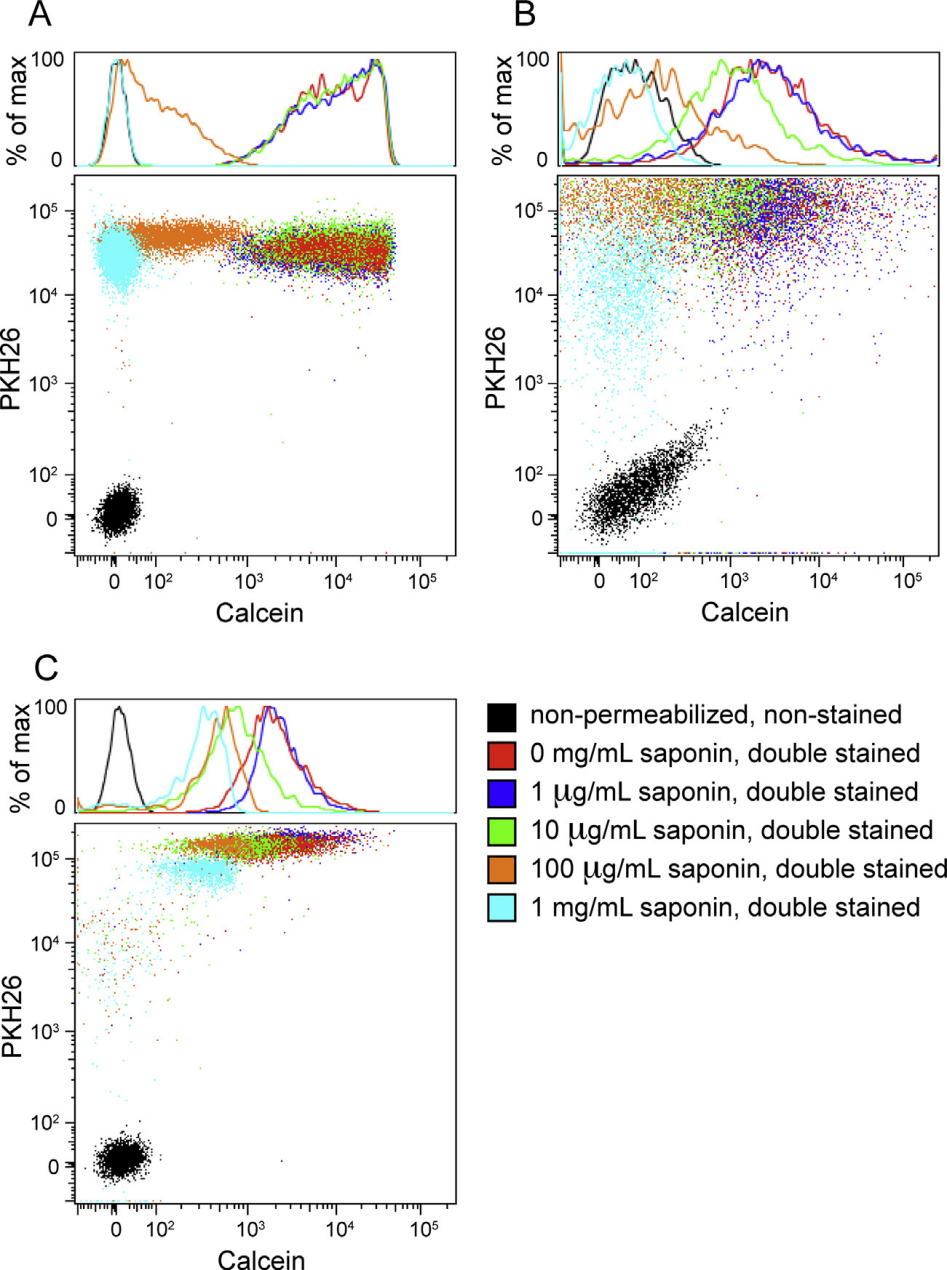
Pre-permeabilization with saponin rendered the permeabilized plasma (A), HAEC (B), and RBC (C) EVs unable to activate or retain calcein AM to the same degree as intact EVs.

Co-staining of non-permeabilized EVs resulted in events that were double positive for calcein AM and PKH26 (Fig. 4, Fig. 5). As expected, with increasing levels of permeabilizing agents, the EVs remained positive for PKH26, indicating that the detected events were lipid membranes, but the calcein fluorescence decreased substantially with increasing permeabilizing agent concentration. This demonstrated that calcein AM labels intact EVs rather than debris.

### Validation Approach 3 – Permeabilization and specific membrane labeling

To further demonstrate that calcein AM labels intact EVs, we modified our first validation strategy by labeling RBC EVs with fluorescently-labeled antibody against the RBC-specific antigen CD235a instead of staining with the general dye, PKH26.

Red blood cell EV pellets were resuspended in 100 μL of filtered PBS before the addition of either anti-CD235a conjugated antibody (PE/Cy7 anti-human CD235a (Glycophorin A); BioLegend, cat. 349112) or the isotype control (PE/Cy7 Mouse IgG2a, κ isotype Ctrl; BioLegend, cat. 400232). After 20 min of incubation at room temperature, the EV suspension was diluted with 1.4 mL of filtered PBS before centrifugation at 16,100 × *g* for 20 min to remove non-bound antibodies. The pellets to be permeabilized were resuspended in 500 μL filtered saponin solution (1 mg/mL) for 20 min at room temperature. All RBC EV pellet suspensions were raised to 1.5 mL with filtered PBS before centrifugation (16,100 × *g*, 20 min). The pellets were resuspended in 100 μL of 10 μM calcein AM and incubated at 37 °C for 20 min. Filtered PBS (200 μL) was added to each of the suspensions before flow cytometry processing (PE/Cy7 excitation_max_496 nm/emission_max_785 nm). Single labeled and non-labeled compensation controls of non-permeabilized EVs were also prepared.

**Fig. 6:**
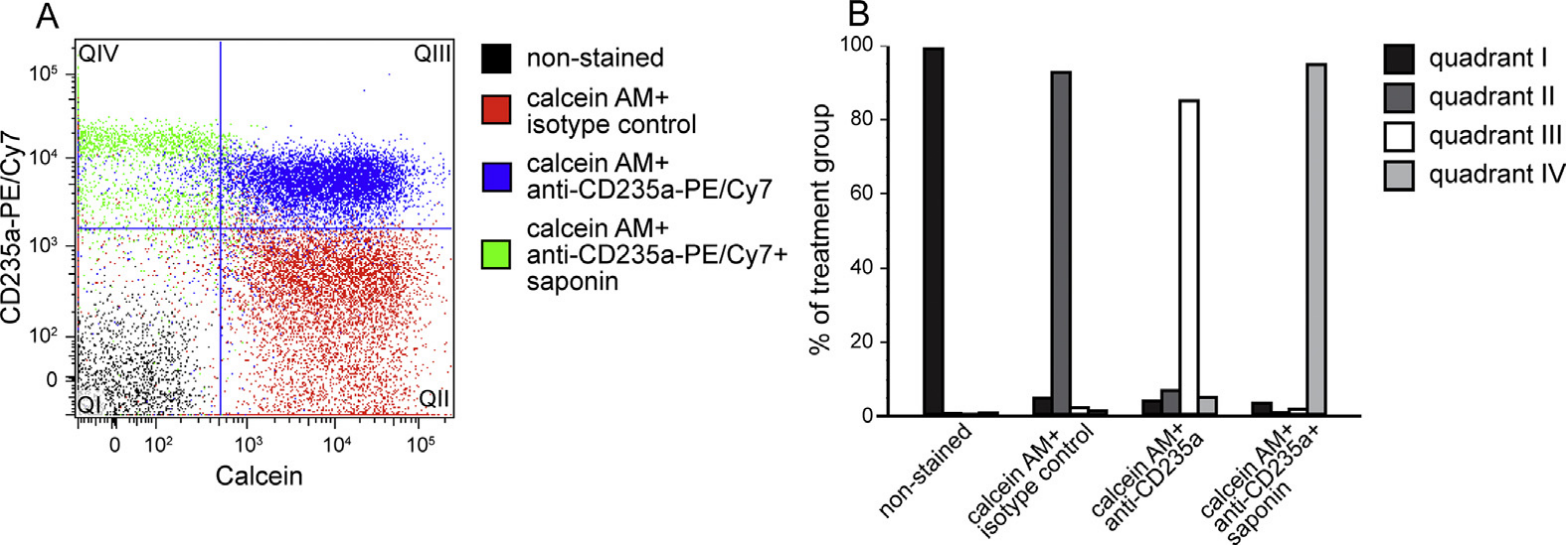
Red blood cell EVs labeled with calcein and anti-CD235a antibody were double positive for both, but lost calcein fluorescence when pre-permeabilized with saponin. Dot plot (A) and quantified events in quadrants (B).

In summary, we found that non-permeabilized EVs were positive for both CD235a and calcein AM labeling (Fig. 6) Upon permeabilization, however, the EVs lacked calcein fluorescence while retaining CD235a labeling. The isotype control-which had not been permeabilized-exhibited calcein positivity but CD235a negativity. These results confirm the previous findings that calcein AM labels intact EVs, rather than lysed EVs or debris.

In our statistical measurement of calcein AM as an indicator for RBC EVs, the sensitivity was 99.1 ± 0.15%, the specificity was 54.3 ± 6.2%, and the calcein CV was 188 ± 2.8% (*n* = 6, mean ± SEM). Furthermore, the method was largely free of systematic or random errors, exhibiting 98.8 ± 0.17% accuracy and 99.8 ± 0.066% precision.

The activation and retention of non-fluorescent calcein AM to the fluorescent calcein in intact EVs – but not permeabilized EVs – indicates that labeling with calcein AM is a suitable method for flow cytometry-based detection of intact EVs released from multiple cells types.
